# The *Drosophila* anterior-posterior axis is polarized by asymmetric myosin activation

**DOI:** 10.1016/j.cub.2021.11.024

**Published:** 2022-01-24

**Authors:** Hélène Doerflinger, Vitaly Zimyanin, Daniel St Johnston

**Affiliations:** 1 The Gurdon Institute and the Department of Genetics, University of Cambridge, Tennis Court Road, Cambridge CB2 1QN, UK

**Keywords:** polarity, microtubule organization, cortical tension, Staufen, oskar mRNA, Slimb, Par-1, myosin pulses

## Abstract

The *Drosophila* anterior-posterior axis is specified at mid-oogenesis when the Par-1 kinase is recruited to the posterior cortex of the oocyte, where it polarizes the microtubule cytoskeleton to define where the axis determinants, *bicoid* and *oskar* mRNAs, localize. This polarity is established in response to an unknown signal from the follicle cells, but how this occurs is unclear. Here we show that the myosin chaperone Unc-45 and non-muscle myosin II (MyoII) are required upstream of Par-1 in polarity establishment. Furthermore, the myosin regulatory light chain (MRLC) is di-phosphorylated at the oocyte posterior in response to the follicle cell signal, inducing longer pulses of myosin contractility at the posterior that may increase cortical tension. Overexpression of MRLC-T21A that cannot be di-phosphorylated or treatment with the myosin light-chain kinase inhibitor ML-7 abolishes Par-1 localization, indicating that the posterior of MRLC di-phosphorylation is essential for both polarity establishment and maintenance. Thus, asymmetric myosin activation polarizes the anterior-posterior axis by recruiting and maintaining Par-1 at the posterior cortex. This raises an intriguing parallel with anterior-posterior axis formation in *C. elegans*, where MyoII also acts upstream of the PAR proteins to establish polarity, but to localize the anterior PAR proteins rather than Par-1.

## Highlights

• *Drosophila* anterior-posterior axis formation requires non-muscle myosin II (MyoII)
• The myosin regulatory light chain (MRLC) is di-phosphorylated at the oocyte posterior
• MRLC-2P induces longer MyoII pulses at the posterior, leading to Par-1 localization
• Inhibiting MRLC di-phosphorylation disrupts polarity establishment and maintenance

## Teaser

The localization of Par-1 to the posterior cortex of the oocyte polarizes the *Drosophila* anterior-posterior axis, but how this occurs was unknown. A new study by Doerflinger et al. shows that Par-1 recruitment requires the di-phosphorylation and activation of myosin II at the posterior, suggesting that cortical tension may act as the polarity cue.

## Introduction

In many organisms, the primary body axis is defined by the polarization of the egg or zygote, generating cellular asymmetries that lead to the localization and segregation of cytoplasmic determinants. This has been extensively characterized in *C. elegans*, where the posterior pole is defined by the site of sperm entry into the unfertilized egg.^1^ Polarity establishment starts when Aurora A associated with the sperm centrosome inhibits myosin activity at the posterior cortex to trigger a contraction of cortical actomyosin toward the anterior.^2^, ^3^, ^4^ The anterior polarity proteins PAR-3, PAR-6, and aPKC, which are initially localized all around the egg membrane, are carried to the anterior by this cortical flow, allowing the posterior polarity factors PAR-2, PAR-1, and Lgl to localize to the posterior cortex.^5^, ^6^, ^7^ After this establishment phase, polarity is maintained by mutual antagonism between the anterior and posterior PAR proteins.^8^, ^9^, ^10^ The localized PAR proteins control spindle orientation and the asymmetric localization of determinants to drive an asymmetric first cell division to produce a large anterior AB cell and a smaller posterior P cell.

Like *C. elegans*, *Drosophila* sets up its anterior-posterior axis at the one-cell stage, but in this case during the development of the oocyte.^11^ Anterior-posterior asymmetry arises in the germarium when the oocyte moves to the posterior end of the 16-cell germline cyst as a result of differential adhesion between the oocyte and the somatic follicle cells.^12^, ^13^, ^14^ The follicle cells at the two ends of the egg chamber subsequently become specified as terminal follicle cells, rather than main-body follicle cells as a result of Unpaired signaling from a pair of polar cells at each pole of the egg chamber.^15^ At stage 6 of oogenesis, the EGF-like ligand Gurken is secreted from the posterior of the oocyte to induce the adjacent terminal follicle cells at this end of the egg chamber to adopt a posterior fate instead of the default anterior fate, and these cells then signal back to the oocyte to induce its polarization along the future anterior-posterior axis.^16^^,^^17^ Despite extensive searches, however, the polarizing signal from the follicle cells has not been identified.^18^

The first sign of the anterior-posterior polarization of the *Drosophila* oocyte is the recruitment of the Par-1 kinase to the posterior cortex at stage 7 of oogenesis, in a process that depends on the actin cytoskeleton.^19^, ^20^, ^21^ At the same time, aPKC and Par-6 are excluded from the posterior cortex, whereas the Par-3 ortholog Baz disappears from the posterior slightly later.^22^ This cortical polarity is then maintained by mutual antagonism between the anterior and posterior PAR proteins, in which Par-1 phosphorylates Baz to exclude it from the posterior cortex and aPKC phosphorylates Par-1 to prevent it from localizing laterally.^22^^,^^23^ Par-1 transduces this cortical polarity to the microtubule cytoskeleton by repressing the formation of non-centrosomal microtubule-organizing centers (ncMTOCs) posteriorly, leading to the formation of a weakly polarized microtubule network that directs the kinesin-dependent transport of the posterior determinant, *oskar* mRNA, to the posterior pole.^24^^,^^25^ Almost nothing is known about how this PAR protein asymmetry is established, except that the ubiquitin ligase Slimb is necessary for the posterior recruitment of Par-1.^26^ Here we report that polarity signaling induces the specific activation of non-muscle myosin II (MyoII) at the posterior of the oocyte and show that this acts upstream of Slimb in the recruitment of Par-1, making it the first sign of polarity establishment identified to date.

## Results

### *unc-45* mutations disrupt anterior-posterior axis formation

**Figure 1:**
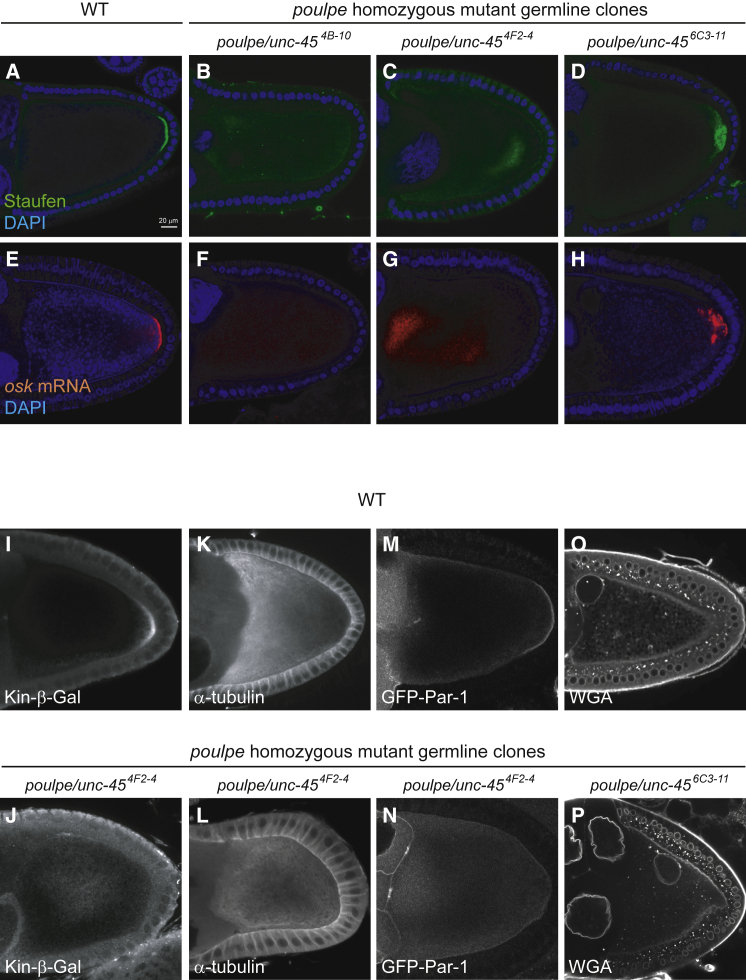
The *poulpe* gene is required for oocyte polarization and acts upstream of Par-1 (A) A confocal image of a wild-type egg chamber showing the localization of Staufen (green) in a crescent at the posterior cortex of the oocyte; DAPI (blue). (B–D) Confocal images showing examples of Staufen localization in *poulpe* homozygous mutant germline clones. Staufen is either diffusely localized (B), localized to the center of the oocyte (C), or forms a diffuse cloud near the posterior pole (D); Staufen (green) and DAPI (blue). (E) A confocal image of a wild-type egg chamber showing the localization of *oskar* mRNA (red) to the posterior cortex of the oocyte; DAPI (blue). (F–H) Confocal images showing examples of *oskar* mRNA localization in *poulpe* homozygous mutant germline clones; *oskar* mRNA (red) and DAPI (blue). (I and J) Kinesin-β-galactosidase localization in wild-type (I) and *poulpe*^4F2-4^ mutant (J) oocytes. The constitutively active kinesin-β-galactosidase (white) localizes to the posterior cortex of the wild-type oocyte by moving toward microtubule plus ends. Kinesin-β-galactosidase localizes to the center of the *poulpe*^42F-4^ mutant oocyte, indicating that the oocyte is not polarized and the plus ends are not focused on the posterior. (K and L) α-tubulin staining showing the microtubule organization in wild-type (K) and *poulpe*^4F2-4^ mutant (L) oocytes. In the wild-type oocyte, the microtubules form an anterior-to-posterior gradient and are most dense along the anterior and lateral cortex, where their stable minus ends are anchored. In the *poulpe*^4F2-4^ mutant, the microtubules are nucleated all around the oocyte cortex, forming a density gradient from the cortex to the center. (M and N) Confocal images showing the localization of UAS-GFP-Par-1 expressed specifically in the germline under mat-tub:Gal4 in wild-type (M) and *poulpe*^4F2-4^ mutant (N) oocytes. Par-1 forms a crescent at the posterior of wild-type oocytes, but is not localized in *poulpe*^4F2-4^ mutants. (O and P) Confocal images showing wheat germ agglutinin (WGA) staining to label the nuclei in a wild-type (O) and *poulpe*^6C3-11^ (P) egg chamber. The oocyte nucleus is anchored at the dorsal/anterior corner of the wild-type oocyte, but is mislocalized to the lateral cortex in the *poulpe*^6C3-11^ mutant. See also Figures S1 and S2.

We identified a complementation group of three alleles that we named “*poulpe*” in a germline clone screen for mutants that disrupt the posterior localization of GFP-Staufen, which acts as a marker for *oskar* mRNA.^27^ In wild-type stage 9–10 egg chambers, Staufen and *oskar* mRNA localize to a well-defined crescent at the posterior of the oocyte, whereas they are often not localized at all, or localize to the center of the oocyte in *poulpe* homozygous mutant germline clones (Figures 1A–1H and S1). In some weaker cases, Staufen and *oskar* mRNA reach the posterior region but form a diffuse cloud rather than a crescent, which is reminiscent of the phenotype seen in mutants that fail to anchor Staufen/*oskar* mRNA complexes once they are localized (Figures 1D, 1H, and S1).

Because the strong *oskar* mRNA mislocalization phenotypes of *poulpe* mutants resemble those seen when the microtubule network is not correctly polarized, we examined the organization of the microtubules by expressing kinesin-βgal, a constitutively active form of kinesin fused to β-galactosidase.^28^ In wild-type ovaries, kinesin-βgal localizes to the posterior of the oocyte at stage 9 by moving along the weakly polarized microtubule network to the region with more plus ends than minus ends, just like *oskar* mRNA (Figure 1I). By contrast, kinesin-βgal forms a cloud in the middle of 80% of *poulpe* mutant oocytes (n > 75), indicating that microtubule plus ends are concentrated in the center (Figure 1M). Wild-type oocytes show an anterior-posterior gradient of microtubule density, with the strongest staining near the anterior/lateral cortex, where the more stable, minus ends reside (Figure 1J). 85% of *poulpe* mutant oocytes (n > 60) show high microtubule staining all around the oocyte cortex, with little signal in the center, suggesting that microtubules are nucleated from the entire cortex (Figure 1N). These microtubule and *oskar* mRNA phenotypes of *poulpe* are very similar to those of strong *par-1* alleles, where ncMTOCs form all around the oocyte cortex and nucleate microtubules that extend toward the center of the oocyte.^19^^,^^25^^,^^29^ We therefore expressed GFP-Par-1 in the germline to examine whether it is recruited normally to the posterior cortex.^21^ There is no obvious Par-1 enrichment at the posterior of *poulpe* mutant oocytes (n > 75), however, indicating that polarity establishment is disrupted upstream of Par-1 localization (Figures 1K and 1O). The oocyte nucleus, which always migrates to and is then anchored at the anterior of wild-type and *par-1* mutant oocytes, is found in the middle of 10% of *poulpe* mutant stage 9 oocytes (n = 70) (Figures 1L and 1P).

Recombination and deficiency mapping placed *poulpe* in the 400-kb region between 84D14 and 84E8-9 and all three alleles failed to complement P{PZ}*unc-4503692*, a lethal P element insertion in the *unc-45* locus.^30^ Sequencing revealed that *poulpe*^*6C3-11*^ and *poulpe*^*4F2-4*^ both have premature stop codons (Q250→Stop; Q573→Stop) in the *unc-45*-coding region and are therefore presumably null alleles, whereas the third allele *poulpe*^*4B4-10*^ is likely to be a rearrangement. Thus, Poulpe corresponds to Unc-45, which is a TPR (tetratricopeptide repeat) and UCS (UNC-45, CRO1, She4p) domain-containing protein that acts as a chaperone for folding and stabilizing myosins.^31^^,^^32^

### MyoII is required for the anterior-posterior polarization of the oocyte

The weak phenotype of some *unc-45* germline clones resembles that of mutants in the single *Drosophila* myosin V, *didum*, in which Staufen and *oskar* mRNA are not anchored at the posterior cortex.^33^^,^^34^ *didum* mutants do not affect the localization of Par-1, however, indicating that Unc-45 must be required for the function of another myosin that plays a role in polarity establishment. Although it is unclear how many of the 14 *Drosophila* myosins require Unc-45, many can be ruled out because their mutants are homozygous viable and fertile or because they are not expressed in the ovary.^35^ We also excluded the myosin VI *jaguar*, because homozygous mutants have no effect on the posterior localization of Staufen (Figure S2).

**Figure 2:**
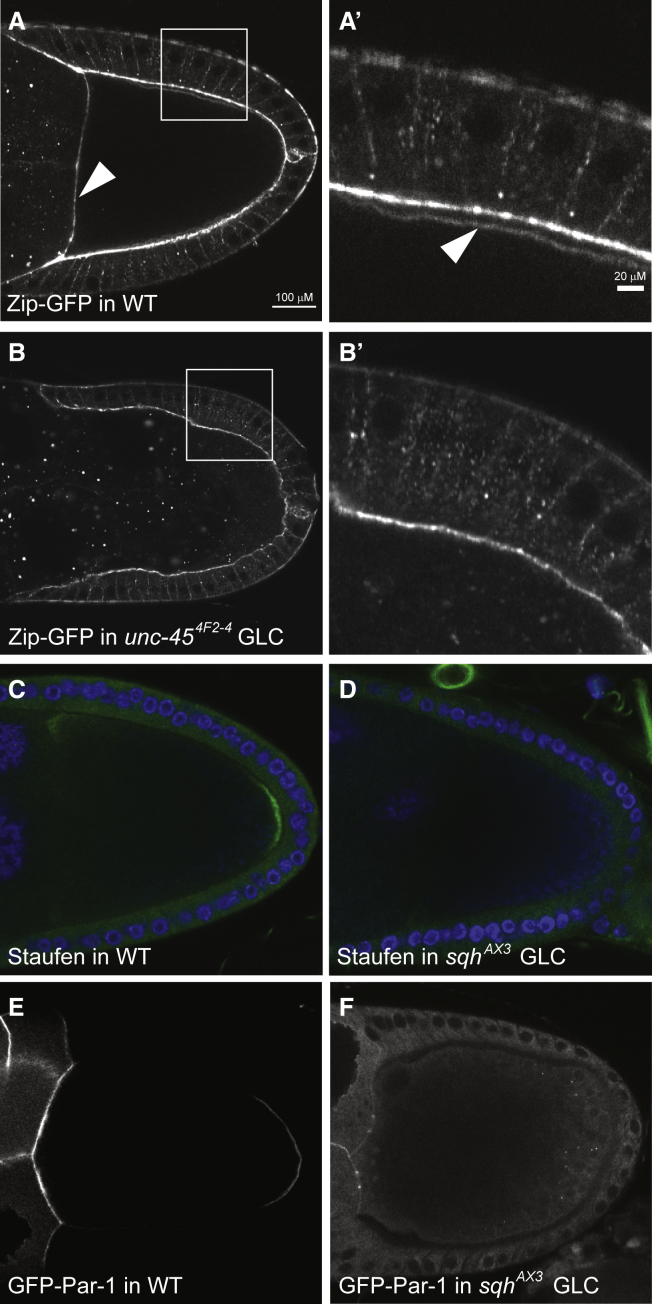
MyoII is chaperoned by Unc-45 and is required for oocyte polarization (A and A′) A confocal image of a wild-type stage 10A egg chamber expressing Zipper-GFP, a protein trap line in the myosin heavy chain. MyoII localizes strongly to the apical side of the follicle cells and more weakly around the cortex of the oocyte beneath (arrowhead in A′). The arrowhead in (A) shows the anterior cortex of the oocyte where it abuts the nurse cells. Scale bars, 100μm (A) and 20μm (A') (B and B′) A confocal image of an *unc-45*^4F2-4^ germline clone expressing Zipper-GFP. MyoII is lost from the oocyte cortex and accumulates in cytoplasmic aggregates, indicating that Unc45 is a MyoII chaperone. (C) Antibody staining of Staufen protein (green) in a wild-type oocyte, showing its localization at the posterior cortex; DAPI (blue). (D) Antibody staining of Staufen protein (green) in a *sqh*^*AX3*^ mutant oocyte. Staufen is not localized posteriorly. (E) A wild-type egg chamber expressing UAS-GFP-Par-1 in the germline under the control of mat-α4tub:Gal4. Par-1-GFP forms a crescent at the posterior cortex of the oocyte. (F) UAS-GFP-Par-1 is not enriched at the posterior cortex of *sqh*^*AX3*^ mutant oocytes.

The most obvious candidate for a myosin involved in polarity establishment is non-muscle MyoII, given its key role in the polarization of the *C. elegans* zygote.^5^^,^^6^ MyoII is a hexamer formed by two molecules of the myosin heavy chain Zipper (Zip) that dimerize through their long coiled-coil tail domains and two copies of the essential light chain and the myosin regulatory light chain (MRLC) Spaghetti squash (Sqh), which both bind to the neck region of each heavy chain.^36^ To test whether Unc-45 is required for the folding and assembly of MyoII, we examined the distribution of endogenous Zipper, using a GFP protein trap insertion.^37^ In wild-type egg chambers, Zipper is strongly enriched at the apical cortex of the follicle cells and localizes at lower levels all around the oocyte cortex. Zip-GFP signal can be resolved from the apical follicle cell signal at high magnification at stage 10A and is most obvious at the nurse cell/oocyte boundary, where there are no follicle cells (Figures 2A and 2A′). This cortical signal is lost in *unc-45* mutant germline clones and Zip-GFP is instead found in aggregates throughout the nurse cell and oocyte cytoplasm (n > 30), indicating that the formation of functional MyoII is impaired (Figures 2B and 2B′). MyoII performs many essential functions in the cell, including driving cytokinesis, and loss-of-function germline clones in the MRLC *sqh* therefore produce a range of defects, such as binucleate nurse cells and germline cysts with the wrong number of cells.^38^ *sqh* mutant germline clones eventually stop producing egg chambers, but sufficient wild-type MRLC perdures to allow some egg chambers to develop until stage 9 of oogenesis and 64% of these fail to localize Staufen to the posterior pole of the oocyte (n = 47) (Figures 2C and 2D). This phenotype does not result from a defect in Staufen/*oskar* mRNA transport, which depends on microtubules rather than actin and is instead caused by a failure to establish anterior-posterior polarity, as shown by the loss of the posterior crescent of Par-1 (Figures 2E and 2F).

**Figure 3:**
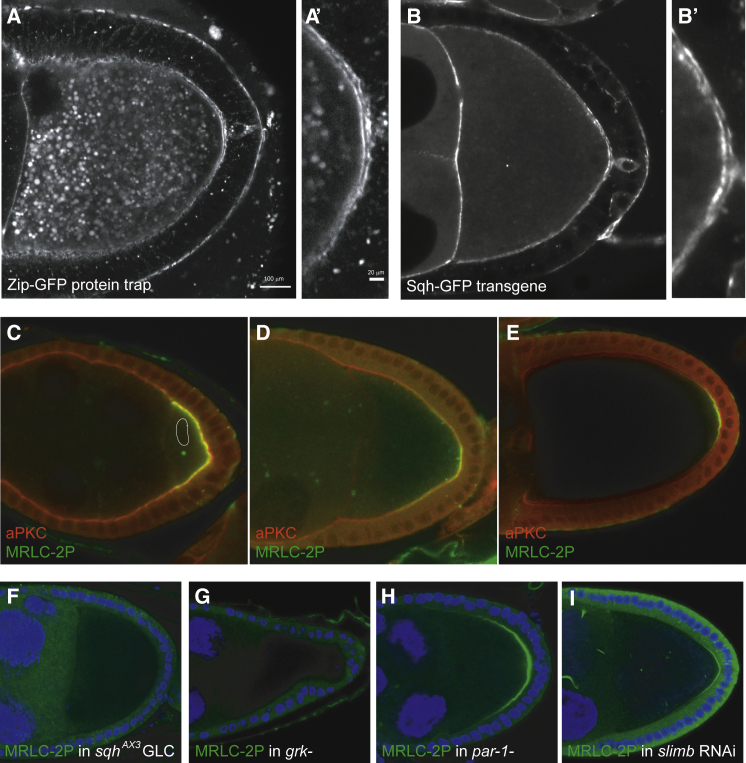
The myosin regulatory light chain is di-phosphorylated at the posterior cortex of the oocyte (A and A′) A confocal image of a living egg chamber expressing Zipper-GFP (MyoII). MyoII is enriched at the posterior cortex of the oocyte. (A′) shows a close-up of the posterior cortex. Scale bars, 100μm (A) and 20μm (A'). (B and B′) A confocal image of a living egg chamber expressing Sqh-GFP (myosin regulatory light chain; MRLC). MRLC is enriched at the posterior cortex of the oocyte (close-up in B′). (C–E) Antibody staining for MRLC-2P (green) in stage 6 (C), stage 9 (D), and stage 10b (E) wild-type egg chambers. The oocyte nucleus is outlined in white in (C). MRLC-2P localizes to the regions of the oocyte cortex that contact the follicle cells at stage 6, becomes restricted to the posterior as the main-body follicle cells migrate to cover the oocyte at stage 9 (D), and persists there until stage 10b (E). (F) MRLC-2P staining in a *sqh*^AX3^ germline clone. No MRLC-2P signal is detected, confirming the specificity of the antibody. (G) MRLC-2P staining in a *grk*^*2B6/2E12*^ mutant egg chamber. The posterior crescent of MRLC-2P is lost, indicating that its formation depends on signaling from the posterior follicle cells. (H) MRLC-2P staining in a *par-1*^*6323/W3*^ mutant egg chamber. The posterior crescent of MRLC-2P forms normally and may be slightly expanded. (I) MRLC-2P staining in an egg chamber expressing *slimb* RNAi in the germline under the control of nos-Gal4. The posterior crescent of MRLC-2P forms normally in the absence of SCF/Slimb function. See also Figure S3.

The requirement for MyoII in oocyte polarization raises the question of whether MyoII itself is polarized. Live imaging of the Zip-GFP protein trap line reveals that MyoII is concentrated in a line at the posterior cortex of the oocyte, whereas the MyoII signal is weaker and more diffuse around the lateral cortex, suggesting that MyoII is asymmetrically activated at the posterior of the oocyte (Figures 3A and 3A′). An identical posterior enrichment is also observed for a Sqh-GFP transgene (Figures 3B and 3B′).

### MRLC is di-phosphorylated at the oocyte posterior

MyoII activity is regulated by the phosphorylation of the conserved threonine 20 and serine 21 of MRLC, which activate its ATPase and motor activities.^39^^,^^40^ We therefore took advantage of specific antibodies that recognize *Drosophila* MRLC (Sqh) that is mono-phosphorylated on just serine 21 (MRLC-1P), the main activating site, or doubly phosphorylated on both serine 21 and threonine 20 (MRLC-2P).^41^ The mono-phosphorylated form of MRLC is enriched at the cortex but shows no obvious asymmetry along the anterior-posterior axis of the oocyte (Figure S3). By contrast, MRLC-2P is strongly enriched at the posterior cortex of the oocyte from stage 7 onward (Figure 3C). This signal initially encompasses the entire region of the oocyte cortex that contacts the follicle cells but, as the main-body follicle cells surrounding the nurse cells migrate posteriorly to cover the oocyte during stage 9, MRLC-2P becomes restricted to a posterior crescent, where the signal persists until late stage 10b (Figures 3D and 3E). The localization pattern of MRLC-2P therefore corresponds to the regions of the oocyte cortex that underlie the posterior terminal population of follicle cells, whereas the timing of the appearance of MRLC-2P coincides with the signal that polarizes the oocyte.

To test whether MRLC di-phosphorylation depends on the polarizing signal from the follicle cells, we examined MRLC-2P in various polarity mutants. As expected, no MRLC-2P is detected in *sqh*^*AX3*^-null mutant germline clones (n > 15), confirming that the signal is specific (Figure 3F). More importantly, MRLC-2P is also completely lost from the posterior cortex of the oocyte in *gurken* mutants (n > 30), which do not specify the posterior follicle cells and lack the polarizing signal (Figure 3G). The posterior MRLC-2P crescent forms normally in *par-1* mutants, however, and may even expand, indicating that MRLC di-phosphorylation is upstream of Par-1 recruitment in the polarity signaling pathway (Figure 3H). The Slimb ubiquitin ligase is the only known factor that acts upstream of Par-1 localization in the oocyte except for the actin cytoskeleton.^26^ Oocytes expressing Slimb RNAi still form the MRLC-2P posterior crescent (Figure 3I). Thus, MRLC is phosphorylated in response to the polarizing signal and lies upstream of Slimb and Par-1 in the signal transduction pathway.

### MRLC di-phosphorylation is required for oocyte polarity

**Figure 4:**
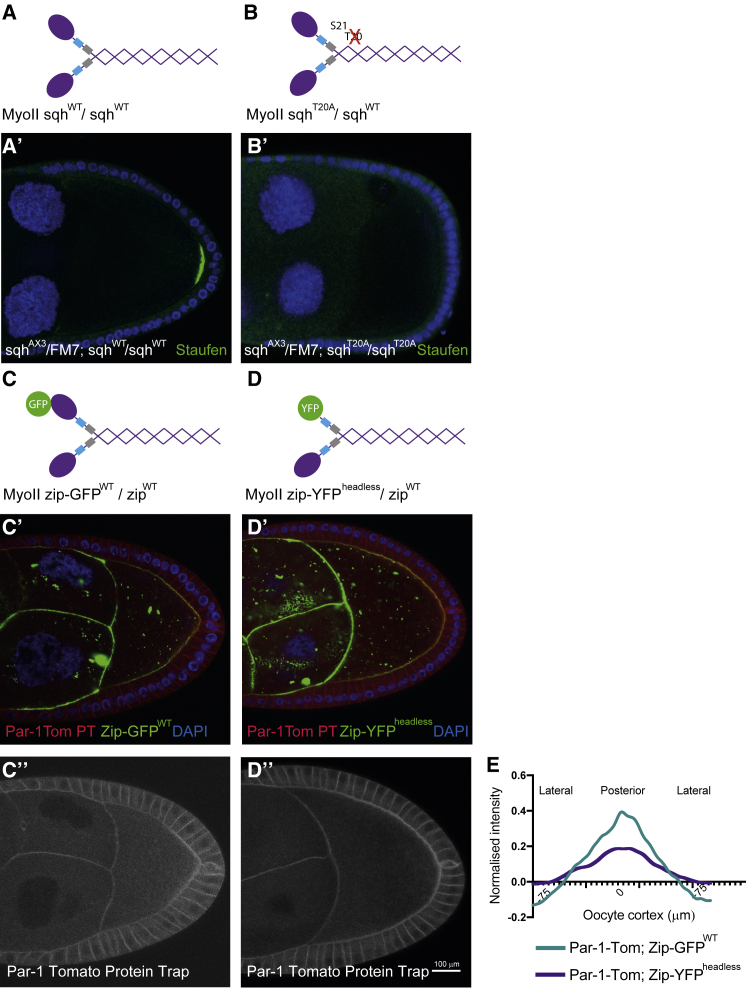
MRLC di-phosphorylation is required for anterior-posterior axis formation (A) A diagram of the structure of wild-type MyoII, with the myosin heavy chain Zipper shown in purple, the essential light chain in light blue, and the MRLC, Sqh in gray. (A′) Staufen staining (green) of a *sqh*^AX3^/FM7; *sqh*^WT^/*sqh*^WT^ egg chamber, expressing one endogenous copy and two transgenic copies of MRLC; DAPI (blue). Staufen localizes normally to the posterior cortex of the oocyte in all cases. (B) A diagram of MyoII containing one copy of wild-type MRLC and one copy of MRLC in which threonine 20 is mutated to alanine (T20A). (B′) Staufen staining of a *sqh*^AX3^/FM7; *sqh*^T20A^/*sqh*^T20A^ egg chamber, expressing one endogenous copy of wild-type MRLC and two transgenic copies of MRLC that cannot be phosphorylated on threonine 20. Staufen fails to localize to the posterior in 48% of stage 9–10 oocytes of this genotype (n = 152). (C) A diagram of wild-type MyoII containing one copy of the myosin heavy chain Zipper, tagged by GFP. (C′ and C′′) A Zipper-GFP^WT^/+ egg chamber showing the localization of MyoII (green), a Par-1-Tomato protein trap insertion (red), and DAPI (blue). (C′′) shows Par-1-Tomato (white), which forms a crescent at the posterior of the oocyte and localizes to the lateral cortex of the follicle cells. (D) A diagram of MyoII containing one copy of Zipper in which the myosin head has been deleted and replaced by YFP (Zipper-YFP^headless^). (D′ and D′′) A Zipper-YFP^headless^/+ egg chamber showing the localization of MyoII (green), a Par-1-Tomato protein trap insertion (red), and DAPI (blue). (D′′) shows Par-1-Tomato (white), which forms a broad and weak crescent at the posterior of the oocyte. Scale bar, 100μm. (E) Quantification of Par-1-Tomato localization along the lateral and posterior oocyte cortex in Zipper-GFP^WT^/+ and Zipper-YFP^headless^/+ oocytes (n = 10 for each group). The posterior pole, marked by the position of the polar follicle cells, lies at 0 μm. The intensity was measured as the ratio of the PAR-1-Tomato signal at the oocyte cortex to the lateral signal in the follicle cells to normalize between different egg chambers.

The discovery that MRLC is specifically di-phosphorylated at the oocyte posterior raises the question of whether this modification is required for oocyte polarity or is just a marker for this process. To test this, we analyzed Sqh-GFP transgenes that cannot be di-phosphorylated because the second phosphorylation site, threonine 20, is mutated to alanine (*sqh*AS).^38^^,^^40^ Although the *sqh*AS transgenes failed to rescue the polarity phenotype of *sqh*^*AX3*^-null mutant germline clones (n > 20), none of the available wild-type *sqh*TS-GFP transgenes could rescue either, presumably because they are not expressed at high enough levels or because the GFP tag impairs their function. We therefore generated new *sqh*TS and *sqh*AS transgenes without the GFP tag and under the endogenous *sqh* promoter. The *sqh*TS transgene appears fully functional because it rescued the fertility of *sqh*^AX3^ germline clones, whereas *sqh*AS did not rescue. Egg chambers from *sqh*^AX3^ germline clones with or without the *sqh*AS transgene showed limited and variable survival to stage 9, which made quantification difficult. We therefore tested whether the *sqh* transgenes have a dominant-negative effect on oocyte polarity when present in two copies in a heterozygous *sqh*^AX3^/+ background. The wild-type *sqh*TS transgene had no effect on oocyte polarity as assayed by the posterior localization of Staufen (n = 25) (Figures 4A and 4A′). By contrast, Staufen was not localized in 48% of *sqh*^AX3^/+ egg chambers overexpressing *sqh*AS (n = 152), indicating that the second phosphorylation of MRLC on threonine 20 plays an essential role in defining the posterior (Figures 4B and 4B′).

The phosphorylation of MRLC on threonine 20 (MRLC-2P) has little effect on the ATPase activity of MyoII *in vitro* in the presence of high concentrations of actin, compared to the form in which just serine 21 is phosphorylated, but does increase ATPase activity when actin is limiting and enhances the speed at which MyoII can translocate F-actin.^40^ Thus, the doubly phosphorylated form of MRLC may generate higher forces and/or faster contractions. To investigate whether MyoII activity is important for oocyte polarity, we examined the effects of overexpressing a headless myosin heavy chain (Zip-YFP^headless^) that can still bind both light chains and form dimers with endogenous MyoII but cannot exert force on actin.^42^ Zip-YFP^headless^ overexpression strongly reduced and broadened the Par-1 crescent (n > 30), whereas wild-type Zip-YFP had no effect on Par-1 recruitment to the posterior (Figures 4C–4E). This suggests that cortical tension plays a role in Par-1 localization, although we cannot rule out the possibility that the headless myosin also disrupts filament formation.

### MRLC-2P induces longer MyoII pulses at the oocyte posterior

**Figure 5:**
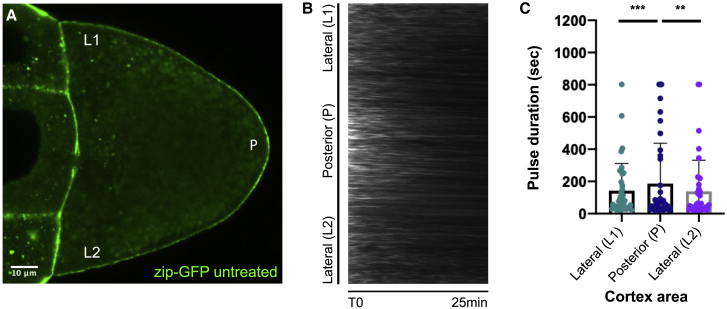
MyoII forms cortical foci that persist longer at the posterior (A) A still image from a movie of a wild-type egg chamber expressing Zipper-GFP in the germline. Zipper-GFP signal is higher at the posterior of the oocyte cortex compared to the lateral sides. Scale bar, 10μm. (B) A kymograph showing the changes in Zipper-GFP levels over time along the oocyte cortex. Zipper-GFP foci remain stationary, indicating that there is no cortical contraction, and oscillate in intensity over time. (C) A graph showing the durations of Zipper-GFP pulses at the lateral and posterior cortex (L1, P, L2). Pulse durations are measured using an automated detection and segmentation algorithm. The pulses at the posterior last twice as long as the lateral pulses. ∗∗, p < 0.01; ∗∗∗, p < 0.002. See also Figure S4.

In *C. elegans*, polarity is established by the contraction of the actomyosin cortex toward the anterior that localizes the anterior PAR proteins by advection.^5^^,^^6^^,^^43^ To test whether a similar mechanism operates in *Drosophila*, we imaged MyoII foci in the oocyte cortex using Zip-GFP. Kymographs tracking the signal along the lateral and posterior cortex over time show that MyoII forms foci that appear and disappear in a way that is reminiscent of the pulsatile contractions observed in various *Drosophila* epithelial cells during morphogenesis.^44^, ^45^, ^46^ Unlike these morphogenetic processes, the myosin foci at the cortex of the oocyte do not undergo large lateral movements, as shown by the nearly horizontal lines produced by each focus in the kymograph (Figures 5B and S4). This suggests that the cortex is constrained, perhaps by connections through microvilli to the overlying follicle cells. More importantly, the MyoII foci are brighter and last longer at the posterior cortex than at the lateral cortex. Quantifying these data reveals that the average pulse duration varies between egg chambers, but the MyoII foci at the posterior cortex always persist more than twice as long as the foci at the lateral cortex (Figures 5C and S4).

### Inhibiting MRLC di-phosphorylation disrupts polarity

**Figure 6:**
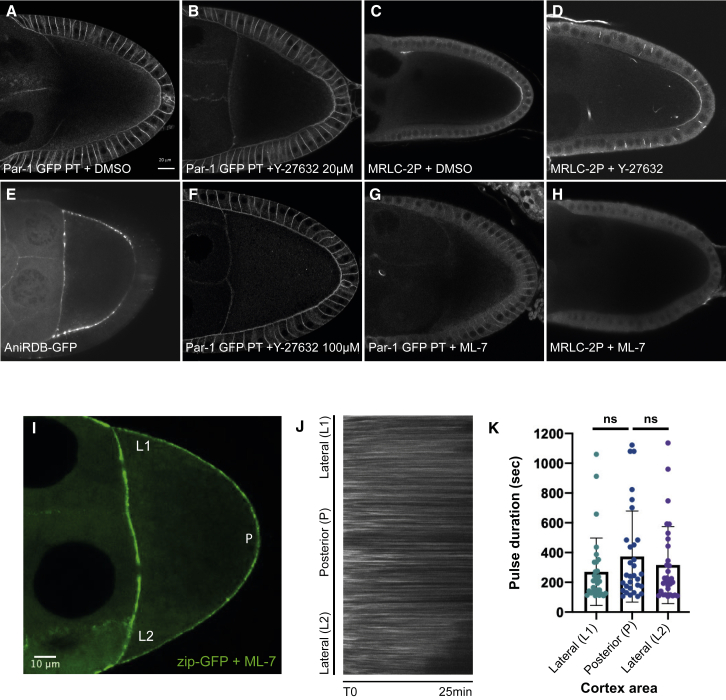
MyoII activation is inhibited by the myosin light-chain kinase inhibitor ML-7 (A) A confocal image of an egg chamber expressing Par-1-GFP from a protein trap insertion after addition of DMSO alone. Par-1-GFP localizes in a crescent at the posterior cortex of the oocyte. Scale bar, 20μm. (B) A confocal image of egg chambers expressing Par-1-GFP after incubation in the Rho kinase inhibitor Y-27632 (20 μm) in DMSO. Y-27632 has no effect on Par-1 localization. (C) A confocal image of MRLC-2P immunostaining in wild-type egg chambers after addition of DMSO alone. MRLC-2P signal forms a crescent at the posterior cortex of the oocyte. (D) A confocal image of MRLC-2P immunostaining in wild-type egg chambers after incubation in the Rho kinase inhibitor Y-27632 (20 μM). Y-27632 has no effect on MRLC-2P localization. (E) A confocal image of an egg chamber expressing the AniRBD-GFP reporter for active Rho-GTP. The AniRBD-GFP signal is lower at the posterior cortex of the oocyte than elsewhere. (F) A confocal image of egg chambers expressing Par-1-GFP after incubation in the Rho kinase inhibitor Y-27632 (100 μM). At this concentration, Par-1-GFP expands around the lateral cortex of the oocyte, presumably because this concentration inhibits aPKC. (G) A confocal image of an egg chamber expressing Par-1-GFP after incubation in the myosin light-chain kinase inhibitor ML-7 (100 μM). Par-1-GFP no longer localizes at the posterior cortex of the oocyte. (H) A confocal image of MRLC-2P immunostaining in wild-type egg chambers after incubation in the myosin light-chain kinase inhibitor ML-7 (100 μM). MRLC is no longer di-phosphorylated at the posterior cortex of the oocyte. (I) A still image from a movie of a wild-type egg chamber expressing Zipper-GFP in the germline 20 min after injection of the myosin light-chain kinase inhibitor ML-7 (100 μM) into the female abdomen. Zipper-GFP signal appears uniform along the oocyte cortex. Scale bar, 10μm (J) A kymograph showing the changes in Zipper-GFP levels over time along the oocyte cortex after treatment with ML-7 (100 μM). Zipper-GFP foci remain stationary and oscillate in intensity over time. (K) A graph showing the duration of Zipper-GFP pulses at the lateral and posterior cortex after ML-7 treatment (L1, P, L2). Pulse durations are measured using an automated detection and segmentation algorithm. The pulses at the posterior are not significantly longer than the lateral pulses (p > 0.05.).

The polarizing cortical contraction in *C. elegans* is a single, transient event that occurs in response to sperm entry early in the first cell cycle. There is no clear morphological sign that indicates when the signal to polarize the *Drosophila* oocyte is produced, however, and we therefore cannot exclude the possibility that there is a cortical contraction that we have not succeeded in visualizing sometime during the 12 or more hours between stages 6 and 9. If this is the case, MyoII activation should be transiently required to establish polarity but would not be needed to maintain it once the PAR proteins are asymmetrically localized. To test this, we examined the effects of acutely inhibiting MRLC kinases after the posterior Par-1 crescent has formed. In many contexts, MyoII is activated by the Rho-dependent kinase Rok, which is inhibited by Y-27632.^47^^,^^48^ However, treating egg chambers with Y-27632 has no effect on posterior Par-1 recruitment or myosin phosphorylation (Figures 6A–6D). Consistent with this, Rho activity, as measured by the AniRBD-GFP reporter, is lower at the posterior cortex of the oocyte than elsewhere (Figure 6E).^49^ Furthermore, treatment with higher concentrations of Y-27632 causes an expansion of the posterior Par-1 crescent rather than a loss, presumably because these concentrations also inhibit aPKC, which phosphorylates Par-1 to exclude it from the lateral cortex (n > 40)^21^^,^^50^ (Figure 6F). This confirms that Y-27632 enters the oocyte efficiently and is active, ruling out Rok as the kinase that phosphorylates Sqh at the posterior. By contrast, exposing egg chambers to ML-7, an inhibitor of the myosin light-chain kinase, leads to a complete loss of posterior Par-1 (76% of the oocytes, n > 50) and Sqh-2P (80% of the oocytes, n > 30) in 15 min (Figures 6G and 6H). The effect of the drug is reversible: after washing the drug away, Par-1 relocalized at the posterior in 75% of the treated oocytes (n > 20). This confirms that MyoII phosphorylation is required to localize Par-1 at the posterior and indicates that this is a continuous requirement.

The discovery that ML-7 specifically inhibits the phosphorylation of Sqh on T21 allowed us to test whether the di-phosphorylation of Sqh is directly responsible for the longer myosin pulses at the posterior. We injected ML-7 into the fly abdomen and imaged endogenous MyoII foci in the oocyte cortex using Zip-GFP. Under these conditions, Zip-GFP is uniformly distributed along the oocyte cortex in 75% of the treated oocytes (n > 40) (Figure 6I). Kymographs tracking the Zip-GFP signal over time show that the MyoII foci still oscillate, but the period of the pulses is not significantly different at the lateral versus posterior cortex (Figures 6J and 6K). Thus, the di-phosphorylation of Sqh increases the duration of actomyosin pulses, presumably leading to higher cortical tension.

## Discussion

Although it was discovered more than 20 years ago that the posterior follicle cells signal to polarize the anterior-posterior axis of the oocyte, almost nothing is known about the nature of this signal or how it is transduced to the oocyte. Here we show that this signaling depends on the myosin chaperone Unc-45 and that this requirement reflects its role in folding non-muscle MyoII. We also find that a key response to the polarizing signal is the di-phosphorylation of MRLC at the posterior of the oocyte, as the appearance of MRLC-2P coincides with where and when the polarizing signal is produced and depends on the specification of the posterior follicle cells by Gurken. More importantly, MRLC di-phosphorylation is required for all subsequent steps in oocyte polarization, because a form of MRLC that can only be mono-phosphorylated on serine 21 acts as a dominant-negative inhibitor of axis formation and blocking the phosphorylation prevents the recruitment of Par-1 to the posterior cortex of the oocyte.

MRLC-2P shows a very different distribution from MRLC-1P in both *Drosophila* and ascidian embryo morphogenesis, but its function *in vivo* has remained unclear.^41^^,^^51^ Our results therefore provide the first example where the di-phosphorylation of MRLC has been demonstrated to play an essential role in development. The second phosphorylation of MRLC on threonine 20 has a negligible effect on MyoII’s ATPase activity *in vitro*, but causes a decrease in the rate of actin translocation and in the rate of apical constriction in the mesoderm of the gastrulating embryo, suggesting that this modification increases the force generated by MyoII.^40^ Because of the clear spatial distribution of MRLC-2P in the *Drosophila* oocyte cortex, our analysis reveals a second effect of the phosphorylation of threonine 20, which is that it doubles the duration of MyoII pulses. This may simply reflect an increase in the time that it takes myosin phosphatase to remove two phosphates, instead of one, or may be due to a more complicated effect on the structure of the myosin hexamer. Nevertheless, this second phosphate presumably allows MyoII to generate more force for longer than the mono-phosphorylated form.

**Figure 7:**
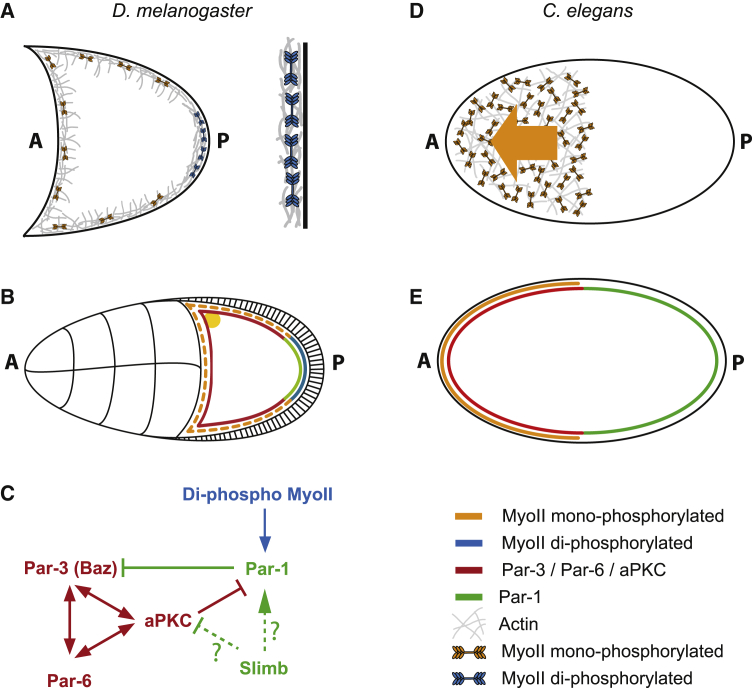
A comparison between the role of MyoII in axis formation in *Drosophila* and *C. elegans* (A) Mono-phosphorylated MyoII is distributed all around the cortex of the *Drosophila* oocyte whereas di-phosphorylated MyoII concentrates at the posterior. Phosphorylated MyoII hexamers form bipolar filaments and generate force between anti-parallel actin filaments, increasing cortical tension. (Mono-phosphorylated MyoII: orange, di-phosphorylated MyoII: blue, Actin (gray). (B) MyoII localises all around the oocyte cortex but is specifically di-phosphorylated at the posterior of the oocyte from stage 6 (blue), leading to the posterior recruitment of Par-1 (green) and the exclusion of aPKC/Par-3/Par-6(red). (Mono-phosphorylated MyoII: orange, di-phosphorylated MyoII: blue, Actin: gray). (C) Proposed signalling pathway for the establishment of AP polarity in the *Drosophila* oocyte. Slimb may act to promote the posterior recruitment of Par-1 in response to the di-phosphorylation of MyoII or could act to exclude the anterior PAR proteins. (D) In the *C.elegans* zygote, MyoII drives a contraction of the actomyosin cortex towards the anterior, which moves the anterior PAR proteins with it by advection, leading to the asymmetric distribution of PAR proteins and the establishment of AP polarity. (MyoII: orange, Actin: gray) (E) The anterior contraction of MyoII in the *C. elegans* zygote results in the localisation of aPKC, PAR-3 and PAR-6 along the anterior cortex and PAR-2 and PAR-1 along the posterior cortex (MyoII: orange, PAR-1 and PAR-2: green, aPKC/PAR-3/PAR-6: red).

The critical function of MRLC-2P in the oocyte is to trigger the recruitment of Par-1 to the posterior cortex, raising the question of how this occurs. The second phosphorylation of MRLC is likely to increase the force generated by MyoII and one would therefore expect to see higher contractility in the posterior oocyte cortex. However, in contrast to the mesoderm, where MRLC-2P increases contraction rates, this does not occur in the oocyte cortex, because there is no lateral movement of MyoII at the posterior or elsewhere. This may be because the actin cortex is different from the mesoderm and cannot contract, possibly because it is denser and rigidly anchored in place through the microvilli that connect to microvilli in the follicle cells. If this is the case, the extra force exerted by MyoII at the posterior should increase the stress on the cortex and on MyoII itself, and this may be the critical change that recruits Par-1 to the posterior (Figures 7A and 7B). For example, MyoII or some other cortical component could act as a tension sensor that exposes a binding site for Par-1, similar to the way in which talin and α-catenin expose binding sites for vinculin when stretched.^52^^,^^53^ This model can explain why the overexpression of headless Zipper disrupts Par-1 localization, because this should result in mixed MyoII hexamers with fewer heads that therefore exert less force.

Any model for Par-1 recruitment must explain the role of Slimb in this process.^26^ Par-1 and the anterior Par-3 (Baz)/Par-6/aPKC complex are mutually antagonistic, with Par-1 excluding Baz from the cortex by phosphorylation and aPKC excluding Par-1 by phosphorylation.^22^^,^^23^ The levels of the anterior polarity factors aPKC and Par-6 are increased in *slimb* mutants, leading to the suggestion that the Slimb/SCF ubiquitin ligase normally targets them for degradation at the posterior, thereby allowing Par-1 to localize there (Figure 7C). Thus, MRLC-2P and the increased tension may promote the SCF-dependent removal of the anterior Par proteins from the posterior. However, it is also possible that Slimb/SCF plays an indirect role in polarity by reducing Par-6/aPKC levels everywhere and thereby setting a threshold for cortical exclusion of Par-1 by aPKC that is overcome specifically at the posterior by its MRLC-2P-dependent recruitment. In this scenario, MyoII-dependent posterior recruitment could protect Par-1 from aPKC phosphorylation in a similar way to that in which PAR-2 has been proposed to protect PAR-1 in *C. elegans*.^54^ Alternatively, Slimb may play a role that is independent of its regulation of aPKC and Par-6. It has recently been shown that the SCF ubiquitin ligase complex regulates the ubiquitylation of Zipper in *Drosophila* auditory organs.^55^ Since Par-1 contains a ubiquitin-binding-associated (UBA) domain, this raises the possibility that SCF ubiquitylates Zipper in response to tension to create a binding site for Par-1. In this context, it is worth noting that the *C. elegans* MyoII heavy chain was first identified in an expression screen for proteins that bind to Par-1 and co-immunoprecipitates with Par-1 from embryos.^56^ Furthermore, Par-1 that cannot be phosphorylated by PKC-3 (*C. elegans* aPKC) concentrates in MyoII foci at the anterior of the *C. elegans* zygote.^57^ However, this co-localization is associated with the auto-inhibition of PAR-1’s kinase activity by its C-terminal KA1 domain. By contrast, deletion of the KA1 domain of *Drosophila* Par-1 has no effect on axis formation and its association with MyoII leads to activation rather than repression.^58^

Anterior-posterior axis formation in *Drosophila* and *C. elegans* is defined by the formation of complementary cortical domains of mutually antagonistic PAR complexes. Our results reveal a further similarity in that myosin activity is required to establish these PAR domains in each case (Figure 7). However, polarity in the worm is established by a myosin-driven contraction of the cortex toward the anterior that localizes the anterior PAR proteins, whereas MyoII activation in the *Drosophila* oocyte localizes Par-1 to the posterior. A second key difference between polarity establishment in worms and flies is that the requirement for MyoII is transient in *C. elegans*, because MyoII contractility is not required to maintain PAR polarity once it is established.^8^ By contrast, myosin activation is continuously required for Par-1 localization in *Drosophila*, because this localization is abolished by ML-7 treatment in oocytes that have already polarized. Thus, there is no evidence for distinct establishment and maintenance phases of oocyte polarization in *Drosophila*. Furthermore, the mutual antagonism between Par-1 and the anterior Par complex is not sufficient to maintain polarity once established in the absence of myosin activation. This difference between worms and flies may reflect the distinct nature of the polarizing cues in each system, because sperm entry in the worm is a one-off event, whereas the posterior follicle cells remain adjacent to the posterior of the *Drosophila* oocyte throughout oogenesis and are therefore in a position to provide the polarizing signal continuously, highlighting the context-dependent relationship between the actomyosin cortex and polarity factors.

Although the role of MyoII in polarity establishment in flies and worms is very different, there is a striking parallel between MyoII’s role in localizing Par-1 posteriorly in the *Drosophila* oocyte and in localizing the cell-fate determinant Miranda basally during the asymmetric cell divisions of the *Drosophila* neuroblasts. Like Par-1, Miranda is excluded from the cortex by aPKC phosphorylation and this was initially thought to be sufficient to explain its basal localization in the neuroblast.^50^^,^^59^ It has recently emerged, however, that aPKC’s main function is to exclude Miranda from the apical and lateral cortex during interphase and that activated MyoII then recruits Miranda basally during mitosis in a process that is inhibited by ML-7.^60^^,^^61^ Thus, Miranda and Par-1 appear to share a common localization mechanism, which may provide a more general paradigm for the role of MyoII in generating cellular asymmetries.

## STAR★Methods

### Key resources table

**Table:** 

REAGENT or RESOURCE	SOURCE	IDENTIFIER
**Antibodies**		

FITC-coupled anti-α-tubulin (1/200)	Sigma	F2168; RRID:AB_476967
Staufen (1/200)	^62^	N/A
SQH2P/MRLC-2P (1/100)	^41^	N/A
SQH1P/MRLC-1P (1/100)	^41^	N/A
SQH2P/MRLC-2P (1/100)	This study. Same peptides as Zhang and Ward^41^	Pocono Rabbit Farm & Laboratory
Alexa conjugated secondary antibodies (1/300)	Thermo Fisher Scientific	N/A
Digoxin Cy3 (1/200)	Jackson Labs	200-162-156; RRID:AB_2339025

**Bacterial and virus strains**		

Bioline silver competent cells	BIOLINE UK LTD	BIO85026

**Chemicals, peptides, and recombinant proteins**		

Wheat germ agglutinin, Texas Red®-X (1/300)	Invitrogen	W21405
Texas Red-X Phalloidin (1/300)	Thermo Fisher Scientific	T7471
VectaShield Mounting Medium with DAPI	Vector Laboratories Ltd	H-1200-10
Myosin light chain kinase inhibitor ML-7	Merck Ltd	475880
ROCK inhibitor Y-27632	Hello BIO	HB2297
Schneider’s Insect medium	Sigma Aldrich Company Ltd	S0146
Insect Cell screened FBS	Thermo Fisher Scientific	SH30070.03I
BSA, fatty acid free	Sigma Aldrich Company Ltd	A4612-1G
Insulin Solution, Human	Sigma Aldrich Company Ltd	I9278-5ML
16% formaldehyde methanol free	Fisher Scientific Worldwide	10751395
Tween 20	Sigma-Aldrich Company Ltd	P1379
tRNA	Roche Applied Science	10109495001
Salmon sperm DNA	Sigma Aldrich Company Ltd	31149-10G-F

**Critical commercial assays**		

QuikChange II XL Site-Directed Mutagenesis kit	Agilent Technologies	200521
Gibson Assembly Master mix	NEW ENGLAND BIOLABS	E2611S
DIG RNA Labeling Mix	Sigma-Aldrich Company Ltd	1127707391

**Experimental models: *Drosophila* stocks**		

*y*^1^*w*^1^ (used as wild type)	Bloomington *Drosophila* Stock Centre	1495
*w* ;; FRT82B *unc-45*^4B4-10^/TM3	^27^ Called poulpe^4B4-10^	N/A
*w* ;; FRT82B *unc-45*^4F2-4^/TM3	^27^ Called poulpe^4F2-4^	N/A
*w* ;; FRT82B *unc-45*^6C3-11^/TM3	^27^ Called poulpe^6C3-11^	N/A
*gurken*^2B6^*b pr cn sca*/CyO	^63^	N/A
*gurken*^2E12^*b* / CyO	^63^	N/A
*w* ; *par-1*^W3^/ CyO	^19^	N/A
*w* ; *par-1*^6323^/Cyo	^19^	N/A
*w* FRT 19A *sqh*^AX3^/FM7	Bloomington *Drosophila* Stock Centre	25712
Df(3R) *jaguar*^322^ /TM3	Bloomington *Drosophila* Stock Centre	8776
*w* ; FRTG13 *didum*^234^/ Cyo	^64^ Called shorty L744	N/A
*w* ;; FRT82B *unc-45*^4B4-10^/TM3	^27^ Called poulpe^4B4-10^	N/A
*y*^1^*sc*^∗^*v*^1^*sev*^21^; slimb RNAi attP	Bloomington *Drosophila* Stock Centre	33986
*w*; UAS-zip-GFP	Bloomington *Drosophila* Stock Centre	80156
*w*; UAS-zip-GFP	Bloomington *Drosophila* Stock Centre	80156
*w*; UAS-zip-YFP^headless^	^42^	N/A
*y w*; *Pin* / Cyo; Kinesin:bgal	^28^	KZ503
*w* ; mat-tub-GFP-Par-1/ CyO	^65^	N/A
*w* ; UASp-GFP:Par-1(N1S)- GFP/CyO	^65^	N/A
Par-1-GFP protein trap / CyO	^66^	N/A
Par-1-Tomato protein trap / CyO	This study	N/A
*w* ; zip-GFP protein trap	Kyoto stock center	115082
*y*^1^*w*^∗^*cv*^1^*sqh*^AX3^; sqh-GFP	Bloomington *Drosophila* Stock Centre	57144
*w* ; sqh^WT^ attP2	This study	N/A
*w* ; sqh^T20A^ attP2	This study	N/A
*w* ; P{tdTomato-0}N1001.5a	St Johnston lab	N/A
*w* ;; ubi-Anillin RDB-GFP	^49^	N/A
*w* ; mat-tub:Gal4	St Johnston lab and Jean-Paul Vincent	N/A
*w* ; mat-tub:Gal4-VP16	St Johnston lab and Jean-Paul Vincent	7063
w ; nanos:Gal4 VP16	^67^	N/A
*w* ;; FRT82B ubi-GFP	Bloomington *Drosophila* Stock Centre	5188
*w* ;; FRT 82B *ovo*D /TM3	Bloomington *Drosophila* Stock Centre	2149
*y w* hs:flp FRT 19A ovoD / C(1)DX	Bloomington *Drosophila* Stock Centre	23880
FRT 19A GFP ; hs:flp	St Johnston lab	N/A
*y v* ;; attP2	Bloomington *Drosophila* Stock Centre	31207
*w*^∗^ ; *Sc*/CyO [Hop]	St Johnston lab	N/A

**Oligonucleotides**		

See Table S1 for oligonucleotides	N/A	N/A

**Recombinant DNA**		

pattB cloning vector	^68^	N/A
pattB -sqhWT	This study	N/A
pattB -sqhT20A	This study	N/A
pBS-*bcd*	St Johnston lab	N/A
pBS-*osk*	St Johnston lab	N/A

**Software and algorithms**		

Olympus Fluoview Version 3.1.	Olympus	https://www.olympus-lifescience.com/en/software/
Fiji Image Processing Software	^69^	https://imagej.net/Fiji
Excel	Microsoft Office	https://www.office.com/
Prism 8	Graphpad	https://www.graphpad.com/scientific-software/prism/

### Resource availability

#### Lead contact

Further information and requests for resources and reagents should be directed to and will be fulfilled by the lead contact, Daniel St Johnston (d.stjohnston@gurdon.cam.ac.uk)

#### Materials availability

All fly stocks and plasmids generated in this study are available on request.

### Experimental model and subject details

All experiments were performed on 3-5 day old *Drosophila melanogaster* females.

Standard procedures were used for *Drosophila* husbandry and experiments. Flies were reared on standard fly food supplemented with dried yeast at 25°C. Heat shocks to induce germline clones were performed at 37°C for 1 h (twice daily) for three days. Flies were kept at 25°C for at least 3 to 5 days after the last heat shock before dissection. UAS-transgenes were expressed using Gal-4 drivers in flies raised at 25°C; adult females were dissected at least 3 to 5 days after they had hatched.

### Method details

#### Drug treatments

Ovaries were incubated in a Schneider’s insect medium solution, 10% FBS and Insulin (1/2000) for 20 min with 20 μM ROCK inhibitor Y-27632 (HelloBio HB2297) or 100 μM myosin light chain kinase inhibitor ML-7 (Merck 475880) and fixed for 20 min in 4% paraformaldehyde and 0.2% Tween-20 in PBS. The ML-7 treatment was tested at 20, 50, 100 and 200μM. Treatment at 100μM gave the strongest effect on the loss of posterior Par-1 enrichment (76%, n = 29). This is similar to the concentration used to disrupt cytokinesis in *Drosophila* spermatocytes.^70^

The reversibility of ML-7 was tested by incubating the ovaries in a Schneider’s insect medium solution, 10% FBS and Insulin (1/2000) for 20 minutes with 100μM ML-7, followed by a 15 min wash in the Schneider’s insect medium and fixed for 20 min in 4% paraformaldehyde and 0.2% Tween 20 in PBS.

#### Immunostaining

Ovaries were fixed for 15 min in 4% formaldehyde and 0.2% Tween 20 in PBS. For phospho-specific antibody immunostainings, a phosphatase inhibitor solution was added to the PBS 0,2% Tween 20 solution. 50X phosphatase inhibitor solution kept at −80°C:

0.105 g NaF (Sigma S79209), 0.540 g B glycerophosphate (Sigma G9422), 0.092 g Na_3_VO_4_ (Sigma 450243), 5.579 g Sodium pyrophosphate decahydrate (Sigma S6422), qsp 50 mL dH2O.

α-tubulin immunostainings: ovaries were fixed 10 min in 10% formaldehyde and 0.2%

Tween 20 in PBS as described by Theurkauf et al.^71^ Ovaries were then blocked in 10% bovine serum albumin (in PBS with 0.2% Tween 20) for at least 1 h at room temperature. Samples were incubated with primary antibodies for at least 3 h in PBS with 0.2% Tween 20 and 10% BSA and were then washed three times in PBS-0.2% Tween 20 for 30 min. They were then incubated in secondary antibodies for at least 3 h in PBS-0.2% Tween 20 and washed again at least 3 times before mounting in Vectashield containing DAPI (Vector laboratories) The concentrations of primary antibodies used are indicated in the Key resources table. Secondary antibodies and Phalloidin were used at 1/300. Incubations with Wheat germ agglutinin (1/300) were performed in PBS with 0.2% Tween 20 for 30 min followed by a 30 min wash.

#### *In situ* hybridizations

Fluorescence *in situ* hybridizations (FISH) were performed according to standard protocols.

Day 1: Ovaries were fixed for 20 min in 4% formaldehyde and PBT 0.2%, and washed three times for 10 min in PBT 0.2%. They were then rinsed in MeOH three times for 5 min and stored in MeOH at −20°C overnight. Day 2: The ovaries were rehydrated in 1:1 MeOH: PBT 0.2% for 10 min, followed by three washes in PBT 0.2% for 10 min. The ovaries were then incubated in 1:1 PBT 0.2%: Pre-hybridization (PH) solution at room temperature for 5 min, followed by an incubation in PH at 65°C for 20 min. The ovaries were incubated in the hybridization solution with 1ul antisense probe overnight at 65°C. Day 3: The ovaries were washed in PH for 30 min at 65°C, followed by a wash in 1:1 PH: PBT 0.2% at 65°C, and three washes in PBT 0.2% at room temperature. The ovaries were then incubated in anti-DIG Cy3 1/200 in PBT 0.2% for 1 hour at room temperature and washed three times for 30 min. The ovaries were mounted in Vectashield and stored at −20°C. All the washes were carried out on a rotator.

The antisense probes for *bcd* and *osk* RNAs were synthesized using the DIG RNA Labeling mix (Sigma 112770739) and the linearized plasmids: pBS-*bcd* cDNA (linearized with HindIII and transcribed with T7) and pBS-*osk* cDNA (linearized with BamHI and transcribed with T3).

PBT 0.2%: 0.2% Tween 20 solution diluted in Phosphate-buffered saline (PBS)

Pre-hybridization solution (PH): For 50ml, 25ml Formamide, 12.5ml 20x saline-sodium citrate buffer (SSC) and 100 μL 100% Tween-20 were mixed, the pH was adjusted to 6.8 with HCl and the volume made up to 50ml with DPEC water.

Hybridization solution: 10 mL of PH was mixed with 20 μL tRNA (20mg/ml) and 10 μL salmon sperm DNA (10mg/ml).

#### Imaging

Imaging was performed using an Olympus IX81 (40 × /1.3 UPlan FLN Oil or 60 × /1.35 UPlanSApo Oil). Images were collected with Olympus Fluoview Ver 3.1. Image processing was performed using Fiji.^69^

#### Molecular biology

To generate the pattB -sqhWT construct, 2.7 kb of sqh genomic DNA was amplified by PCR with the oligos H472 and H473 (see Table S1) and inserted in the PhiC31 integration pattB cloning vector^68^ digested with XbaI-BamHI using the Gibson assembly method (Gibson Assembly Master mix NEB). To generate the pattB -sqhT20A construct, we used the Q5 Site Directed Mutagenesis kit (NEB) to generate the sqhT20A mutation in the pattB -sqhWT construct using oligos H334 and H335 (See Table S1). The pattB-sqhWT and pattB-sqhT20 constructs were injected into y v;;attP2 line *Drosophila* embryos^72^ to generate transgenic lines. Adults were crossed to TM3,Sb for balancing.

The par-1-Tomato protein trap was generated by replacing the GFP tag of the par-1-GFP protein trap by the Tomato tag using the P swap technique.^73^ A Tomato transposon donor line in the appropriate reading frame (PIGP3{tdTomato-1}) was crossed with the Par1-GFP protein line together with the Hop transposase line. Larvae from this cross were screened with a Leica MZ16 fluorescent microscope and individual red fluorescent larvae were picked into a fresh vial. Adults were crossed to CyO for balancing.

#### Analysis of MyoII pulses

The distribution of MyoII along the oocyte cortex was analyzed by recording the fluorescence from a UAS-Zipper-GFP line expressed in the germline. The flies were dissected under Voltalef 10S oil and imaged at 40x magnification on a confocal microscope. The Zipper-GFP signal was imaged with a 40x 1.3 NA objective once every 15 s for 25 min with a pixel size of 0.198 μm. Fiji was used to generate kymographs of the MyoII foci over time and to quantify the duration of the MyoII pulses.^69^ The durations of MyoII pulses signal were automatically measured by tracking adjacent strong intensity pixels in the kymograph. 25 consecutive measurements were pooled to determine the average time of MyoII expression at the lateral and posterior cortex of the oocyte.

### Quantification and statistical analysis

Statistical analyses were performed using Prism and statistical significances were calculated by ANOVA (8.4.3 GraphPad Software). Details of sample sizes are provided in the text. Unless stated otherwise in the figure legends, images comparing the same signal across conditions are scaled equivalently.

## Data Availability

Microscopy data reported in this paper will be shared by the lead contact upon request. This paper did not generate any code. Any additional information required to reanalyze the data reported in this paper is available from the lead contact upon request.

## Supplemental information

Document S1. Figures S1–S4 and Table S1

Document S2. Article plus supplemental information
