# Supplementary material for: The Drosophila anterior-posterior axis is polarized by asymmetric myosin activation
Source: Curr Biol. 2022 Jan 24;32(2):374–385.e4. doi: 10.1016/j.cub.2021.11.024 (PMC8791603; doi:10.1016/j.cub.2021.11.024)
Supplement: Document S1. Figures S1–S4 and Table S1 [file mmc1.pdf]

**Current Biology, Volume *32***

## Supplemental Information

# The *Drosophila* anterior-posterior axis is polarized by asymmetric myosin activation

**Hélène Doerflinger, Vitaly Zimyanin, and Daniel St Johnston**

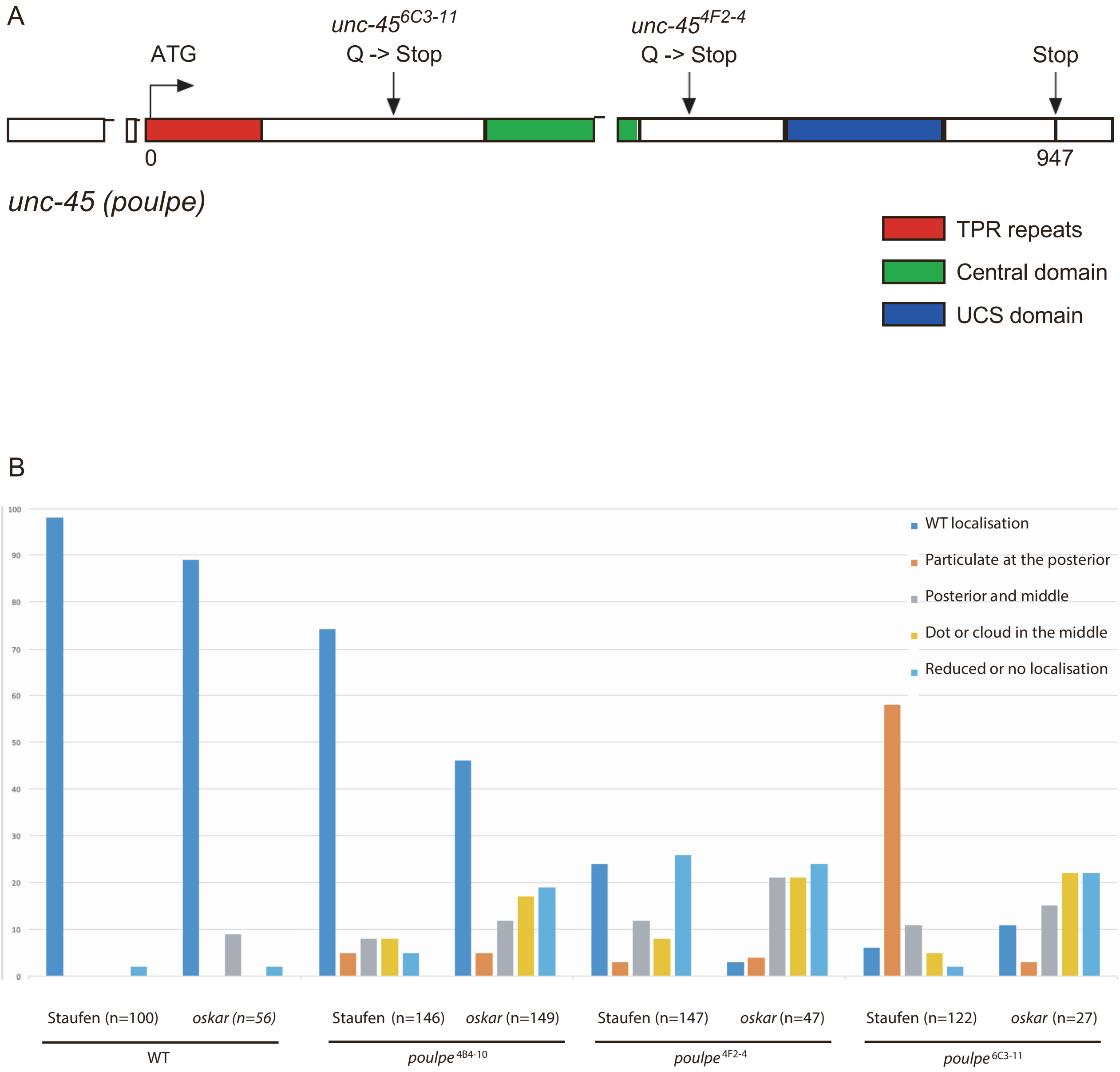
A
unc-45 6C3-11
Q -> Stop
unc-45 4F2-4
Q -> Stop
ATG
Stop
0
947
unc-45 (poulpe)
TPR repeats
Central domain
UCS domain
B
100
90
80
70
60
50
40
30
20
10
0
WT localisation
Particulate at the posterior
Posterior and middle
Dot or cloud in the middle
Reduced or no localisation
Staufen (n=100)
oskar (n=56)
Staufen (n=146)
oskar (n=149)
Staufen (n=147)
oskar (n=47)
Staufen (n=122)
oskar (n=27)
WT
poulpe 4B4-10
poulpe 4F2-4
poulpe 6C3-11

**Figure S1**

**Characterisation of the *unc-45* mutations and their phenotypes in the oocyte, Related to Figure 1**

(A) Schematic representation of the *unc-45* (*poulpe*) coding region with the relative positions of the nonsense mutations in *poulpe [6C31-11]* and *poulpe [4F2-4]*. TPR: tetratricopeptide repeat (red), Central domain (green) and UCS: UNC-45, CRO1, She4p (blue)

(B) Quantification of Staufen and *oskar* mRNA localisation in WT oocytes and in oocytes mutant for the *poulpe [4B4-10], [4F2-4]* and *[6C3-11]*. Wild type (dark blue); Particulate at the posterior (orange); Posterior and middle (grey); Dot or cloud in the middle (yellow); Reduced or no localisation (light blue).

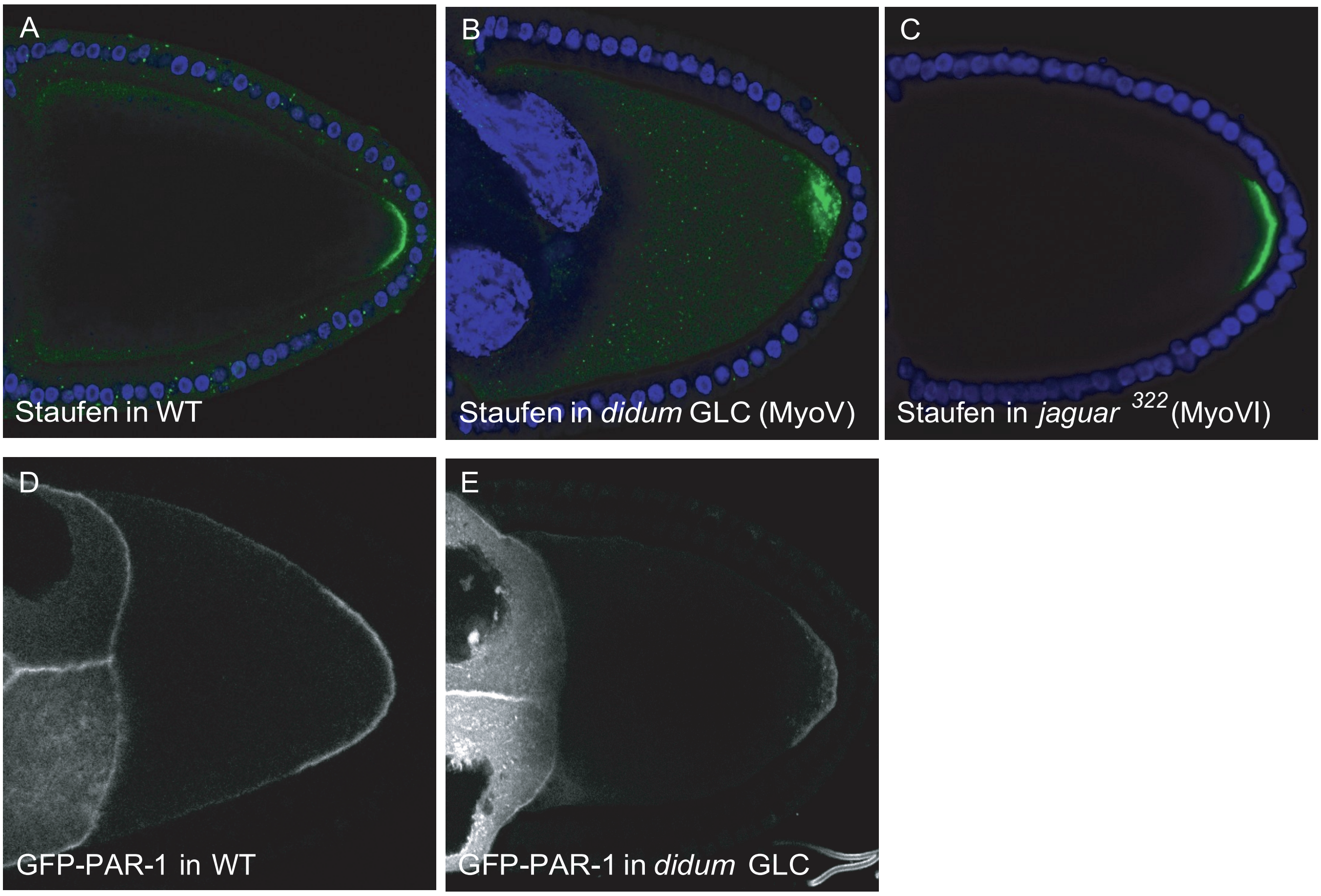
A
Staufen in WT
B
Staufen in *didum* GLC (MyoV)
C
Staufen in *jaguar* 322 (MyoVI)
D
GFP-PAR-1 in WT
E
GFP-PAR-1 in *didum* GLC

**Figure S2**

**Myosin V acts downstream of Par-1, Related to Figure 2**

(A) A confocal image of a wild-type egg chamber showing the localisation of Staufen (green) in a crescent at the posterior cortex of the oocyte; DAPI (blue).

(B) A confocal image showing Staufen localisation in a *didum*$^{234}$ (Myosin V) mutant oocyte derived from a germline clone. Staufen forms a diffuse cloud near the posterior pole; Staufen (green) and DAPI (blue).

(C) A confocal image showing Staufen localisation in a *jaguar*$^{322}$ (Myosin VI) homozygous mutant oocyte. Staufen forms a wild-type crescent at the posterior pole; Staufen (green) and DAPI (blue).

(D-E) Confocal images showing Par-1 GFP in a crescent at the posterior cortex of a wild type oocyte (D) and in a *didum*$^{234}$ homozygous mutant germline clone.

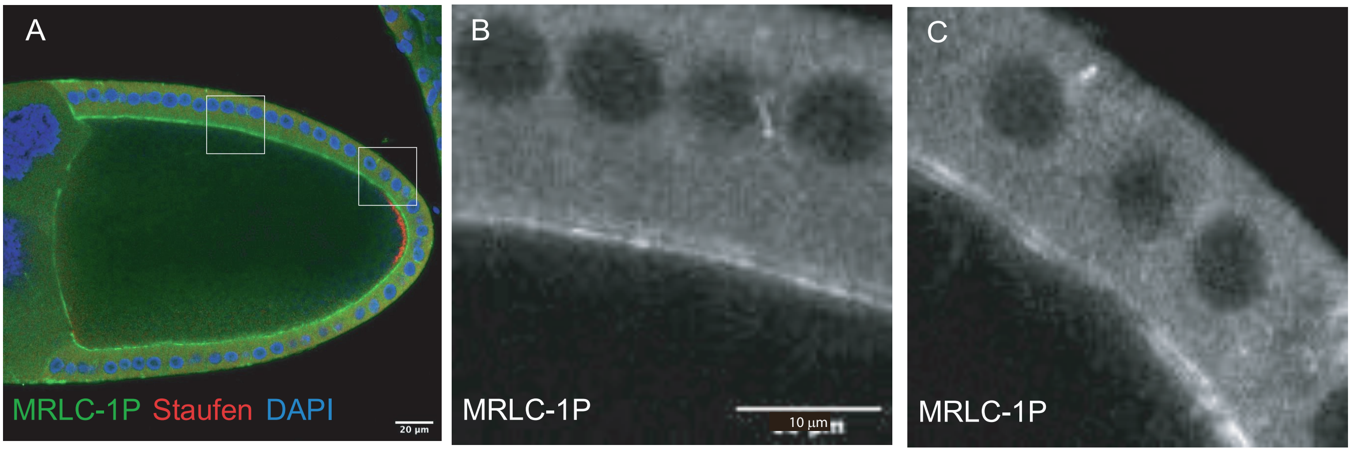
A
MRLC-1P Staufen DAPI
20 µm
B
MRLC-1P
10 µm
C
MRLC-1P

**Figure S3**

**MRLC-1P localises uniformly along the oocyte cortex, Related to Figure 3**

(A-C) Antibody staining of mono-phosphorylated myosin regulatory light chain (MRLC-1P) (green) and Staufen (red) in a wild-type oocyte; DAPI (blue). MRLC-1P is distributed uniformly along the oocyte cortex. White squares indicate the localisation of the magnifications shown in (B; lateral cortex) and (C; posterior cortex).

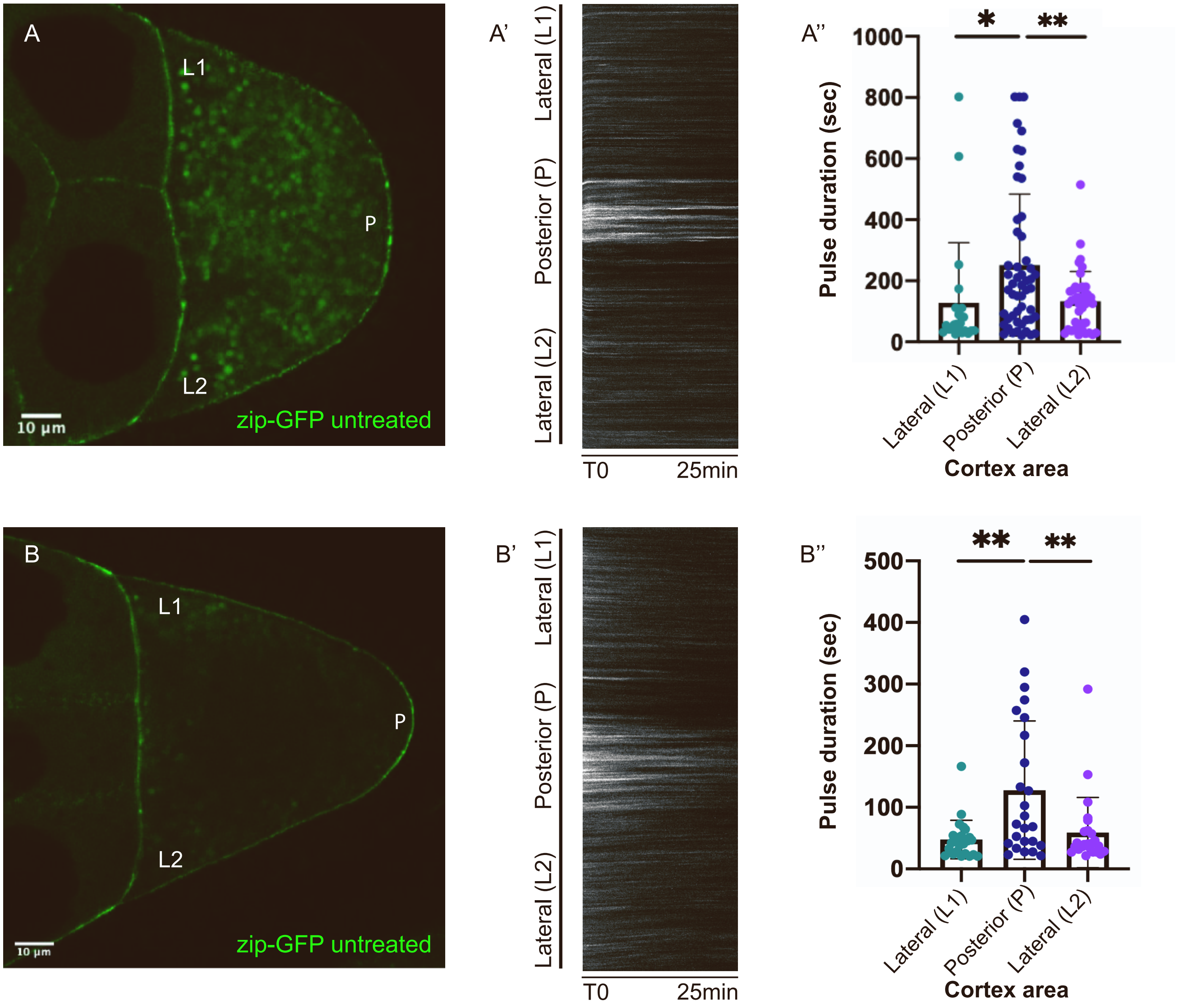
A
L1
P
L2
10 µm
zip-GFP untreated
A'
Lateral (L1)
Posterior (P)
Lateral (L2)
T0
25min
A''
*
**
1000
800
600
400
200
0
Pulse duration (sec)
Lateral (L1)
Posterior (P)
Lateral (L2)
Cortex area
B
L1
P
L2
10 µm
zip-GFP untreated
B'
Lateral (L1)
Posterior (P)
Lateral (L2)
T0
25min
B''
**
**
500
400
300
200
100
0
Pulse duration (sec)
Lateral (L1)
Posterior (P)
Lateral (L2)
Cortex area

**Figure S4**
**MyoII forms cortical foci that persist longer at the posterior – Additional examples, Related to Figure 4**

(A-B) Still images from movie of Zipper-GFP localisation in a stage 9 oocytes

(A'-B') Kymographs showing the changes in Zipper-GFP levels over time along the top and bottom lateral cortices and the posterior cortex between them. Zipper-GFP foci oscillate in intensity over time, but remain stationary, indicating that there is no cortical contraction.

(A''-B'') Graphs showing the durations of Zipper-GFP pulses at the lateral and posterior cortex (L1, P, L2). Pulse durations are measured using an automated detection and segmentation algorithm. The pulses at the posterior last twice as long as the lateral pulses.

| Name | Sequence |
|---|---|
| **poulpe/unc-45 alleles sequencing** | |
| VZ50 | ATATAGAACAGGGCAGCAGCCG |
| VZ51 | AACACATCGGGGATTTCCGC |
| VZ52 | ATCTTTGAAAGCGGCTCC |
| VZ53 | TTGAACGCATTGTCTGGG |
| VZ54 | GCTCTACCACTCCAAGAACG |
| VZ55 | TGCTAAGCAACACATACCG |
| VZ56 | ACCCTCGCCATCGTTGTTATC |
| VZ57 | CCAAATCACATTAGGCCGCAG |
| VZ58 | TTCTGTTGGTCTCCCTTTG |
| VZ59 | ACTTCCTCGCTGTTGATGG |
| VZ60 | GTCGTATCACAATCCCCAC |
| VZ61 | CAAGATGTCCACGATGTAGC |
| VZ64 | TCTCACTTCCTTGGTTCCTGTCC |
| VZ65 | ATGAAGGCGGGATTCTACGGAG |
| VZ66 | CTGCGAATGGCAACTGTTG |
| **Sqh transgene constructs** | |
| H472 pattB -sqhWT | TAACTTCGTATAATGTATGCTATACGAAGTTATGCTAGCG cgagtcttttggacatgaactcattagt |
| H473 pattB -sqhWT | CTTCGAGACCGTGACCTACATCGTCGACACTAGTGGATCT ggggctgccagactgtg |
| H334 pattB -sqhT20A | CCAACGCGCCGCCTCCAATGT |
| H335 pattB -sqhT20A | GCGCGCTTCTTGGTGGTG |

Table S1 – Oligonucleotides
